# Molecular Mechanisms Related to Oxidative Stress in Retinitis Pigmentosa

**DOI:** 10.3390/antiox10060848

**Published:** 2021-05-26

**Authors:** Carla Enrica Gallenga, Maria Lonardi, Sofia Pacetti, Sara Silvia Violanti, Paolo Tassinari, Francesco Di Virgilio, Mauro Tognon, Paolo Perri

**Affiliations:** 1 Department of Medical Sciences, University of Ferrara, 44121 Ferrara, Italy; gllcln@unife.it (C.E.G.); fdv@unife.it (F.D.V.); tgm@unife.it (M.T.); 2 Department of Specialized Surgery, Section of Ophthalmology, Sant’Anna University Hospital, 44121 Ferrara, Italy; maria.lonardi@unife.it (M.L.); sofia.pacetti@unife.it (S.P.); paolo.tassinari@unife.it (P.T.); 3 Department of Head and Neck, Section of Ophthalmology, San Paolo Hospital, 17100 Savona, Italy; sarasilvia.violanti@unife.it; 4 Department of Neuroscience and Rehabilitation, Section of Ophthalmology, University of Ferrara, 44121 Ferrara, Italy

**Keywords:** inflammation, retinitis pigmentosa, oxidative stress, P2X7R, micro-RNA, long non-coding RNA

## Abstract

Retinitis pigmentosa (RP) is an inherited retinopathy. Nevertheless, non-genetic biological factors play a central role in its pathogenesis and progression, including inflammation, autophagy and oxidative stress. The retina is particularly affected by oxidative stress due to its high metabolic rate and oxygen consumption as well as photosensitizer molecules inside the photoreceptors being constantly subjected to light/oxidative stress, which induces accumulation of ROS in RPE, caused by damaged photoreceptor’s daily recycling. Oxidative DNA damage is a key regulator of microglial activation and photoreceptor degeneration in RP, as well as mutations in endogenous antioxidant pathways involved in DNA repair, oxidative stress protection and activation of antioxidant enzymes (*MUTYH*, *CERKL* and *GLO1* genes, respectively). Moreover, exposure to oxidative stress alters the expression of micro-RNA (miRNAs) and of long non-codingRNA (lncRNAs), which might be implicated in RP etiopathogenesis and progression, modifying gene expression and cellular response to oxidative stress. The upregulation of the *P2X7* receptor (P2X7R) also seems to be involved, causing pro-inflammatory cytokines and ROS release by macrophages and microglia, contributing to neuroinflammatory and neurodegenerative progression in RP. The multiple pathways analysed demonstrate that oxidative microglial activation may trigger the vicious cycle of non-resolved neuroinflammation and degeneration, suggesting that microglia may be a key therapy target of oxidative stress in RP.

## 1. Introduction

Retinitis pigmentosa (RP) is an inherited retinal degeneration caused by a collection of different genetic mutations, most of which are related to the progressive loss of photoreceptors (both rod and cone cells) and retinal pigmented epithelium (RPE) dysfunction [1]. This significant genetic burden is also associated with an important phenotypic heterogeneity, due to different penetrance, expressivity [2] and interaction with oxidative stress factors.

Cellular homeostasis requires a balance between oxidative species and antioxidant defence mechanisms. An excessive production of reactive oxygen species (ROS) and free radicals leads to oxidative stress, causing cellular dysfunction, necrosis, apoptosis or autophagic cell death [3,4].

The retina is particularly affected by oxidative stress due to different factors: high oxygen consumption related to a high metabolic demand, the presence of photoreceptors, which contain photosensitizer molecules such as polyunsaturated fatty acids, and the accumulation of ROS in RPE, as an effect of damaged photoreceptor’s daily recycling [5].

Due to high metabolic activity, the retinal tissue has developed multiple defence mechanisms against oxidative damage. Oxidative stress (OS) is related to the activation of specific molecular pathways, such as *PERK* (PKR-like endoplasmic reticulum kinase) and *IRE1* (inositol-requiring enzyme 1), that promote the transcription of genes encoding antioxidant enzymes. However, the chronic activation of some of these pathways causes mitochondrial damage in the long term, with intracellular accumulation of ROS resulting in retinal damage [6]. 

On the other side, inactivating mutations of genes involved in endogenous antioxidant defences leads to a rapid progression of the disease. In fact, in this review, we also analysed the role of *GLO1* (Glyoxalase 1) and *CERKL* (ceramide-kinase like): mutations in these genes increase the sensitivity of retinal tissue to oxidative damage, resulting in cellular apoptosis and retinal neuro-degeneration due to the lack of resilience of the retinal tissue exposed to oxidative stress [7]. 

Among the defence mechanisms, the role of autophagy was also highlighted, prevalent in the retinal ganglion cells (RGC), which have a dual effect: reduction of intracellular ROS levels and mitochondrial support [8,9].

In a study conducted by Rodriguez-Muela et al. using a rd10 mouse model, it was shown that the calcium overload, the activation of calpain, the increase in cathepsin B activity, the reduced colocalization of cathepsin B with lysosomal markers and the reduction in the autophagosomal marker LC3-II (lipidated form of LC3-microtubule-associated protein 1A/1B-light chain 3) expression lead to an increase in permeability of the lysosomal membrane. All these changes in cellular activity, which occur before the death of photoreceptors, are markers of lysosomal dysfunction and down-regulation of autophagic activity [10].

Indeed, recent studies have discovered that the exposure to oxidative stress determines altered expression of micro-RNA (miRNAs) and of long non-coding RNA (lncRNAs) that might be implicated in the etiopathogenesis and progression of RP, since they alter gene expression along with the cellular response to oxidative stress [11].

This review focuses on the molecular mechanisms related to oxidative stress occurring in RP. These mechanisms play a central role in RP pathogenesis and progression. 

### Association between RP-Causative Mutations and Activation of UPR, PERK and IRE1 as a Response to Oxidative Stress 

In response to environmental stress, such as OS, the translation of stress-inducible transcripts encoding heat shock proteins (HSP) is enhanced [3]. HSP perform chaperone functions by ensuring the correct folding of new proteins or refolding proteins that are damaged by OS. 

A pathway that is responsible for cell translation reprogramming upon stress is *PERK*, a protein that is active in response to the accumulation of misfolded proteins [4].

Genetic mutations that were discovered to be causative of RP are involved in misfolding of transmembrane proteins implicated in photoreception and phototransduction. The accumulation of misfolded or unfolded proteins causes an increase in ROS, enhancing oxidative stress and the activation of an unfolded protein response (UPR), *PERK* and *IRE1* pathways in photoreceptors cells. These pathways, involved in endogenous antioxidant defence, if chronically stimulated, determine the activation of pro-apoptotic programs associated with oxidative stress, pro-inflammatory signalling, dysfunctional autophagy, free cytosolic Ca^2+^ overload and an altered protein synthesis rate in the retina [4], leading to retinal degeneration [5] (Table 1). 

## 2. Mutations in Endogenous Antioxidant Pathways: *MUTYH*, *CERKL* and *GLO1*

### 2.1. Role of 8-Oxoguanine and MUTYH in RP

One of the most prevalent genotoxic lesions is 8-oxoguanine (8-oxoG), and it is generated in DNA attacked by ROS [12]. MUTYH (mutY DNA glycosylase) plays an important role in the maintenance of genomic integrity through the activation of pathways involved in DNA repair. It removes adenine (A) from 8-oxoG:A mispairs, through the base excision repair (BER) pathway, preventing mutations in the genome [13].

MUTYH deficiency prevents single-strand breaks (SSBs) formation and cell death under oxidative stress [14,15].

However, under severe oxidative DNA damage, the excessive activation of MUTYH leads to formation of SSBs of DNA, causing disturbed homeostasis and cell death [16].

MUTYH-mediated BER is critical to promote retinal degeneration and inflammation in RP. As demonstrated by Oka et al. in a rd 10 mice model, it occurs through two different pathways, (i) mitochondrial SSBs mediate calpain activation and (ii) nuclear SSBs induce poly (ADP-ribose) polymerase (PARP) activation [17].

The accumulation of 8-oxoG in the rd10 mouse retina is attenuated by hMTH1 over-expression and the activation of MUTYH, which leads to a reduction of rod and cone photoreceptor cell death [18], as well as microgliosis in rd10 mice [17]. Oxidized nucleic acids are increased in the photoreceptor layer but also in immune cells, such as microglial cells and macrophages, which infiltrate outer retinal regions. These findings suggest that oxidative DNA damage in the outer nuclear layer (ONL) of rd10 mice is derived from the oxidized nucleotide pool that occurs in photoreceptor cells and in other non-neuronal proliferating cells in the ONL, such as microglia, that may incorporate oxidized nucleic acids into their nuclear DNA during retinal degeneration, suggesting that these inflammatory cells could be an alternative source of ROS in RP [19].

In microglia, nuclear accumulation of 8-oxoG is associated with PARP activation occurring before the peak of photoreceptor degeneration; thereafter, it expands to the photoreceptor nuclei along with microglial activation [17]. Therefore, oxidative microglial activation may trigger the vicious cycle of non-resolved neuroinflammation and degeneration in RP as it happens in the brain [20], suggesting that the microglia, and especially the MUTYH-SSBs-PARP pathway, may be a key target of oxidative stress in RP (Table 1).

### 2.2. Oxidative Stress in RP: The Role of CERKL

*CERKL* (ceramide-kinase like) is a gene involved in the oxidative stress protection [21]. The precise function of *CERKL* is yet to be determined, but many studies show that it is implicated in the cellular response to oxidative stress and may play a role in protecting cells against stress injury [22,23]. The name of the gene stems from the diacylglycerol kinase domain, which shares homology with ceramide kinases.

Ceramide is a chore sphingolipid (SL), a precursor of other bioactive and complex SLs lipid secondary messengers that control cell status [24,25] and plays a key role in stress-induced apoptosis [24].

To avoid entering apoptosis, induced by the increase of ceramide, cells activate enzymatic pathways involved in its clearance. In this context, the phosphorylation of ceramide, by ceramide kinase, produces a protective effect against apoptosis. Overexpression of *CERK-L* isoforms protects cells from apoptosis induced by oxidative stress [21].

Mutations in *CERKL*, that have been reported to cause distinct RP [26,27], with characteristic macular and peripheral lesions and other cone-rod dystrophy (CRD), support the concept that failure in the endogenous mechanisms to overcome oxidative stress leads to an accelerated progression of retinal neurodegeneration (Table 1).

### 2.3. Glyoxalase 1 (GLO1) Related Genes and Pathways

Glyoxalase 1 (GLO1) is a ubiquitous cellular enzyme involved in detoxification of cytotoxic products of glycolysis, such as α-oxoaldehydes, methylglyoxal (MG), glyoxal (GO) and 3-deoxyglucosone (3-DG). In detail, GLO1 metabolizes MG and prevents MG-induced damage. An excess of MG inactivates antioxidant enzymes, such as glutathione peroxidase and superoxide dismutase (SOD) enzymes, impairing the degradation of MG, determining a positive feedback loop [28].

These cytotoxic products’ levels are increased in cells undergoing hyperglycemic metabolism, such as the RPE cells and photoreceptors, representing the principal source of intra- and extracellular advanced glycation end products (AGEs) [29]. High levels of AGEs, in addition to ROS, determine hyperinflammation and permanent tissue damage [30]. Intracellular AGE precursors, such as MG and GO, can also modify and inhibit the function of important enzymes, such as glyceraldehyde-3-phosphate dehydrogenase (GAPDH) and GLO1 [31].

AGEs exert their injuring effects by direct glycation of intracellular proteins and lipids, by the activation of cell signalling pathways through their binding to cellular receptors and the modulation of gene expression [32].

In the retina, AGEs could alter intra- and extracellular protein structure, increase inflammation and oxidative stress and, therefore, promote vascular dysfunction [33]. The retinal AGEs deposition could cause an upregulation of vascular endothelial growth factor (VEGF), a downregulation of pigment epithelium-derived factor (PEDF) and, eventually, a significant disruption of the inner blood–retinal barrier (iBRB) [34]. Additionally, increased advanced lipoxidation end-products (ALEs) accumulation was also detected in the outer retina. This portion contains photoreceptors, mainly rich in polyunsaturated fatty acids and therefore highly susceptible to lipid peroxidation [35].

In the retina, RPE cells exert the activity of protection by oxidative stress [36,37]. Numerous studies have validated the presence of high levels of ROS and AGEs in RPE, which are able to alter transduction pathways and gene expression [38].

GLO1 mutations, which are part of the endogenous detoxification system regulating ROS and AGE levels, contribute to the accumulation of AGEs in the retina, playing a role in RP pathogenesis [7,39] (Table 1).

Recent studies have identified 22 GLO1-related genes, with their related pathways, to be involved in a complex network of intracellular biochemical mechanisms that might be associated with RP onset and progression. Such pathways include microtubules and actin assembly, ubiquitin-proteasome activity, RE and Golgi integrity, vesicular trafficking, transcriptional and translational control, glycolytic metabolism regulation and glycosylation modifications [40].

In fact, the global down-expression of these genes, excluding the upregulation of *AUTS2* (cytoplasmatic activator of transcription and developmental regulator) and *ANKH* (progressive ankylosis protein homolog), could mostly lead to impairment in cell polarity and adhesion, through the alteration of actin filament structure and activity, with the final result being an increase in RPE apoptosis. Some of these genes are also involved in cell death through the dysregulation of energy metabolism or the translation machinery (*SIK3*, *IPO3* and *MRPS33*).

The main mutations involving genes that compromise cellular metabolism, leading to RPE dysfunction and photoreceptor damage, which could accelerate retinal degeneration in RP, are explained below and summarized in Table 2.

#### 2.3.1. AUTS2

Cytoplasmic activator of transcription and developmental regulator, *AUTS2*, is involved in the activation of the Rho family small GTPase Rac1. This pathway aims to control neuronal migration and neurite extension through the coordination of actin polymerization and microtubule dynamics. In the nucleus, *AUTS2* functions as a transcriptional activator of many target genes, together with the polycomb complex 1 (PRC1) [41].

#### 2.3.2. ARHGAP21 and PTPN13

Rho GTPase activating protein 21 (*ARHGAP21*) is involved in many pathways, both extra and intracellular, such as the inhibition of cell migration and proliferation, cell polarity, cell adhesion, Golgi regulation and positioning, intracellular trafficking and glucose homeostasis [42].

Protein tyrosine phosphatase non-receptor type 13 (*PTPN13*) is a tyrosine phosphatase which mediates phosphoinositide 3-kinase (PI3K) signalling through dephosphorylation of phosphoinositide-3-kinase regulatory subunit 2 (PIK3R2) [43]. In addition, *PTPN13* plays a relevant role in the same extracellular mechanism as *ARHGAP21*. Therefore, it controls negative apoptotic signalling [44] as the antagonist of Rho GTPase A, SLIT-ROBO Rho GTPase activating protein 1 (*SRGAP1*) [45].

#### 2.3.3. FMNL2

Formin-like protein 2-*FMNL2* is involved in the modulation of actin polymerization and organization of the cytoskeleton [46] through the regulation of contractility during epithelial junction maturation [47].

#### 2.3.4. UBC, MYO18A, EPS15, ANKH

Ubiquitin C (*UBC*), Myosin XVIIIA (*MYO18A*), epidermal growth factor receptor pathway substrate 15 (*EPS15*) and *ANKH* inorganic pyrophosphate transport regulator are also involved in intracellular transport processes, and the dysregulation of these genes could influence vesicular trafficking of RPE cells, essential for photoreceptor outer segment (POS) renewal, visual cycle intermediate regeneration and avoiding AGE accumulation.

Specifically, *MYO18A* is involved in actin’s retrograde treadmilling and its transport from focal adhesions to the leading edge [48]. *ANKH* regulates trans-Golgi network trafficking and endocytosis [49], and finally, *EPS15*, with its encoded protein, is involved in clathrin-coated pit maturation, including invagination or budding and cell growth regulation [50,51].

#### 2.3.5. *RFFL*, *FBXW2*, *CAND1*

These three genes are involved in the ubiquitin-proteasome system activity, and their down-regulation could lead to ER stress, inducing the accumulation of misfolded proteins and AGEs.

*RFFL* (ring finger and FYVE-like domain containing E3 ubiquitin protein ligase /rififylin) is an E3 ubiquitin-protein ligase which plays an important role in the extrinsic pathway of apoptosis, modulating cellular death domain receptors [52,53].

*FBXW2* (F-box/WD repeat-containing protein 2) enhances the ubiquitylation and degradation of ß-catenin, overexpressed in the WNT/ß-catenin pathway during inflammatory processes.

*CAND1* (Cullin-associated NEDD8-dissociated protein 1) controls the tubules’ elongation and retraction, thereby regulating the tubular endoplasmic reticulum network [54].

The down-expression of *FBXW2* and *CAND1* also plays a role in ROS production and cell death, altering cellular respiration processes through the impairment of glycolytic metabolism in RPE cells.

#### 2.3.6. *SIK3*

*SIK* family kinase 3 (*SIK3*) gene encodes for a specific kinase that up-regulates mTOR, CREB signalling and cholesterol biosynthesis, correlating with retinoid metabolism and melanogenesis [55].

The down-expression of *SIK3* could cause a defect in energy metabolism, decreasing mitochondrial respiration and up-regulating autophagy in order to remove dysfunctional cellular components, leading to cellular antioxidant mechanisms’ impairment [56,57].

#### 2.3.7. *IPO7*

Importin 7 (*IPO7*) functions as a receptor for nuclear localization signals (NLS) and promotes translocation of import substrates through the nuclear pore complex (NPC). Its down-regulation could trigger p53-dependent growth arrest, ribosomal biogenesis stress and nucleolar morphology changes [58].

#### 2.3.8. *MRPS33*, *MORC4*, *MCPH1*, *NFIA*, *CTIF* and *LMBRD1*

Down-expression of mitochondrial ribosomal protein S33 (*MRPS33*) could damage mitochondrial protein synthesis [59]. MORC family CW-type zinc finger 4 (*MORC4*) and Microcephalin 1 (*MCPH1*) dysregulation arrests DNA damage repair and causes apoptosis [60,61]. Reduced levels of nuclear Factor I A (*NFIA*) impair mitotic exit and cell differentiation [62]. Cap-binding complex-dependent translation initiation factor (*CTIF*) down-expression alters the intermodulation between translation and the aggresome-autophagy pathway, compromising mRNA and protein’s quality control [63]. Lastly, LMBR1 domain containing 1 (*LMBRD1*) down-regulation could affect the transport and metabolism of cobalamin, decreasing distribution of cyanocobalamin (vitamin B12) from the blood to the retina [64,65].

## 3. miRNA Altered Expression and Oxidative Stress:

miRNAs represent a group of short non-coding RNAs that are involved in transcript degradation or translational inhibition of their target mRNAs, whose function is to regulate post-transcriptional gene expression [66]. They are key regulators of many important biological processes [67], controlling specific pathways by targeting networks of functionally correlated genes.

Alterations of miRNA expression, due to mutations in either the miRNA itself or its target genes, could lead to several pathological conditions.

There is much evidence to support the role of miRNAs in normal retinal development and functions [68]: deletion of specific retina-enriched miRNAs has relevant effects on the development of retinal diseases, such as RP [69].

Luigi Donato et al. investigated the complexity of human retina miRNome (murine miRNA transcriptome), analysing data from human RPE cell transcriptomes.

Due to its specific proteins, RPE has many functions, including (i) the regeneration of outer segments of photoreceptors by phagocytizing the spent discs, (ii) regulating the trafficking of nutrients and waste products to and from the retina, (iii) protecting the outer retina from excessive high-energy light and the subsequent light-generated reactive oxygen species and (iv) maintaining retinal homeostasis thanks to the release of diffusible factors.

As a result of all this metabolic activity, RPE cells are very susceptible to oxidative stress [36,70]. Furthermore, RPE cells contain a significant number of mitochondria that are the principal cause of ROS production and removal inside the cell [70]. OS plays a critical role in the etiopathogenesis of RP [71] and leads to pathobiological changes in RPE cells [72] determining outer blood–retina barrier dysfunction [73], inhibition of processing of photoreceptor outer segments by RPE [74], expression of transforming growth factor-β2 [75] and synthesis alterations of extracellular matrix components [76]. All these changes lead to increased RPE apoptosis [77] and senescence changes [72,78].

In a recent paper [11], authors compared changes in the expression of miRNAs obtained from whole transcriptome analyses between two groups of RPE cells, one untreated and the other exposed to the oxidant agent oxidized low-density lipoprotein (oxLDL). In the treated samples, 23 miRNAs revealed altered expression, targeting genes involved in several biochemical pathways, many of which were associated with RP. Moreover, five RP causative genes (*KLHL7*, *RDH11*, *CERKL*, *AIPL1* and *USH1G*) emerged as already confirmed targets of five altered miRNAs (*hsa-miR-1307*, *hsa-miR-3064*, *hsa-miR-4709*, *hsa-miR-3615* and *hsa-miR-637*), suggesting a connection between induced oxidative stress and RP development and progression.

The finding of new regulative functions of miRNAs, and especially their altered expression induced by OS in RPE, should lead to the discovery of alternative mechanism responsible of the etiopathogenesis and progression of RP.

## 4. Role of Long Non-Codingrna

Long non-coding RNAs (lncRNAs) are untranslated transcripts that regulate many biological processes through epigenetic modifications, RNA splicing, mRNA decay and mRNA translation [79], acting as scaffolds for chromatin-modifying complexes [80].

Recent studies have highlighted the close connection between OS, the biochemical pathway involved in RP pathogenesis, and lncRNAs differential expression [81,82] in RPE metabolism.

In cells such as RPE, the high metabolic demand determines the up-regulation of DNA metabolic processes that cause an increased rDNA silencing, due to chromatin-associated lncRNAs, that leads to cellular growth alterations and RPE cell death [83].

In this process, lncRNAs have a key role, as it is well-known that they are involved in DNA metabolic processes: lncRNAs up- or down-regulation could alter gene expression, along with cellular responses to OS, which is one of the most important pathways involved in RP.

Considering DNA damage, two different lncRNAs are likely induced and overexpressed: MNX-AS1 and MIR31HG.

They interact with Cyclin D1 mRNA, whose encoding gene is already known to transcribe a specific lncRNA involved in DNA damage condition, acting as transcription repressors [84].

Oxidative stress also determines changes in glucose metabolism. Specifically, the dysregulation of two lnc-RNAs that are implicated in bioenergetic reactions related to glucose, BDNF-AS and TUG1 [85], were discovered to induce RPE apoptosis [86,87].

Moreover, high glucose levels influence the synthesis of IGF-1, PEDF, AGEs and their receptors (RAGE) that determine OS and inflammatory reactions, leading to retinal degeneration [88].

Additionally, other deregulated lncRNAs connected with insulin-related pathways, such as ARF, AKT1 [89], CRNDE, CYTOR CAP1 and ACACA [90,91], were discovered to induce RPE cell apoptosis [37].

Considering lipid metabolism and homeostasis, RPE cells present intracellular signalling pathways whose gene transcription is regulated by the peroxisome proliferator-activated receptor (PPAR) [92].

In the study by Donato et al. [37], three lncRNAs (AC007283.1, AC012442.2 and AC089983) were identified as being involved in fatty acids biosynthesis and metabolism. In particular, the down-expression of AC007283.1 and AC012442.2, along with the over-expression AC089983.1, could alter the gene expression and lipid metabolism regulation by PPAR-alfa.

Additionally, several down-regulated lncRNAs, such as AC004943.2 and AC007036.3, were found to interact with various miRNAs involved in fatty acid metabolism and biosynthesis, leading to the impairment of the integrity and functionality of lipidic retinal structures [37].

Lastly, the involvement of numerous lncRNAs was also identified in protein metabolism. It is well-known that protein’s misfolding, including those related to retinal survival and vision process, like rhodopsin, determine the disruptions of cellular protein homeostasis [93] and could lead to cell death.

Two clusters made of dysregulated lncRNAs and their interactors/host genes were detected to be involved in cellular amide metabolism [37]. Among them, the up-regulation of PTEN-induced putative kinase protein 1 (*PINK1*) antisense RNA and the downregulation of FMRP translational regulator 1 (*FMR1*)-IT1 sense intronic and vimentin (*VIM*) antisense 1 RNA were found to be particularly interesting.

*PINK1* regulates mitochondrial damage, promotes mitophagy and protects cells from death and apoptosis, especially during high glucose-mediated regulation of RPE [91], reflecting the apoptosis status of RPE.

*FMR1-IT1* and *VIM-AS1* are related to synaptogenesis, intracellular trafficking and cellular stability [94,95], representing the attempt of RPE cells to boost the production of vital proteins.

## 5. P2X7 Receptor and Inflammation in RP

The P2X7 receptor (P2X7R) is an ATP-gated ion channel expressed by immune and inflammatory cells and is over expressed during inflammation and by stressed or dying cells involved in innate and adaptive immune responses. It is recognized as a potent trigger of ROS production, and its over-stimulation leads to the impairment of mitochondrial metabolism, caspase activation as well as apoptosis induction [96,97]. Its stimulation also induces ATP release by means of a membrane pore formation or in association with pannexin hemichannels, activating the NLRP3 (NLR family pyrin domain-containing 3) inflammasome that induces the maturation and release of pro-inflammatory cytokines (IL-1β and IL-18) and the production of ROS, released by macrophages and microglia, contributing to the progression of neuroinflammatory and neurodegenerative diseases [98,99].

The expression of P2X7R was showed in several components of the retinal layers: not only on photoreceptor cells, but also on retinal ganglion cell, amacrine and horizontal cells, microglia and Müller glial cells, astrocytes and pericytes as well as RPE cells [100].

An upregulation of P2x7R mRNA was demonstrated within the retina of an RP mouse model when photoreceptor degeneration occurred. Furthermore, intravitreal administration of ATP in this animal model caused photoreceptor cell death and loss of function due to the activation of P2x7R. As a demonstration, this process can be delayed by intravitreal injection of a P2x7 receptor antagonist in rd1 mouse models [101].

## 6. The Dual Role of Microglia in RP: Between Neurotoxicity and Neuroprotection

We have already analysed two mechanisms that determine an activation of microglia, involving MUTYH and P2X7 receptors. The excessive activation of MUTYH determines nuclear accumulation of 8-oxoG, which is the most prevalent genotoxic lesion, and the PARP pathway as a consequence of accumulation of oxidized nucleic acids, while the upregulation of P2X7R is induced by stressed retinal cells that release extracellular ATP, leading to microglial chemotaxis and activation [100].

Herein, we report in detail the molecular mechanism underlying the activation of microglia and the role of these cells in the development of RP.

The microglia is composed of immune cells implicated in neuronal homeostasis and innate immune defences. Microglia’s cells are activated in response to the altered physiology of mutation-bearing photoreceptors [102], inducing the production of proinflammatory cytokines and chemokines [103,104,105].

A study conducted on a rd1 mouse line demonstrates that in the early stages of disease, there is a persistent upregulation of microglial markers, such as *Tmem119*, *C1qa*, *TNF*, *Il1a* and *Il1b*, that act as inflammatory factors, determining neurotoxicity [106,107,108,109]. A rd1 mouse line treated with PLX5622, a potent colony-stimulator factor 1 receptor (CSF1R) inhibitor that eliminates microglial population, did not show an increase of these factors.

However, it is known that the role of microglia in the development of RP is dictated by a balance between neurotoxic and neuroprotective/neurotrophic influences.

In fact, the activation of retinal microglia induces the expression of neurotrophic factors in Muller cells, exerting neuroprotection in degenerating photoreceptors [110]. Furthermore, it has been demonstrated that overexpression of TGF-beta, an anti-inflammatory cytokine that has a modulatory effect on the action of microglia [111], exercises a protective effect on the degeneration of the cones.

More specifically, through RNA-seq, it has been highlighted that the effects are explicit through post-translational modifications of the proteome not detectable using RNA-seq.

Inhibition of this neuroprotective pathway, induced by the expression of TGF-beta, determines microglial activation, resulting in degenerative changes in retinal tissue given by the expression of proinflammatory cytokines [112].

Finally, the last mechanism concerns the interaction between microglia and complement activation, specifically between the central complement component (C3) and the microglia-expressed receptor (CR3) [113].

An increased C3 expression was found in microglia translocated in ONL after rod degeneration, with a concomitant opsonization of the degenerating photoreceptor by iC3b, a product of C3 activation. This complement activation is therefore a microglial response aimed at phagocytosis of the apoptotic photoreceptor and the restoration of homeostasis. When both C3 and CR3 are deficient, it causes an increased proinflammatory cytokine expression, accumulation of apoptotic cells and neurodegeneration [114].

These findings could lead to other possible immunomodulatory therapy opportunities.

## 7. Conclusions

**Figure 1:**
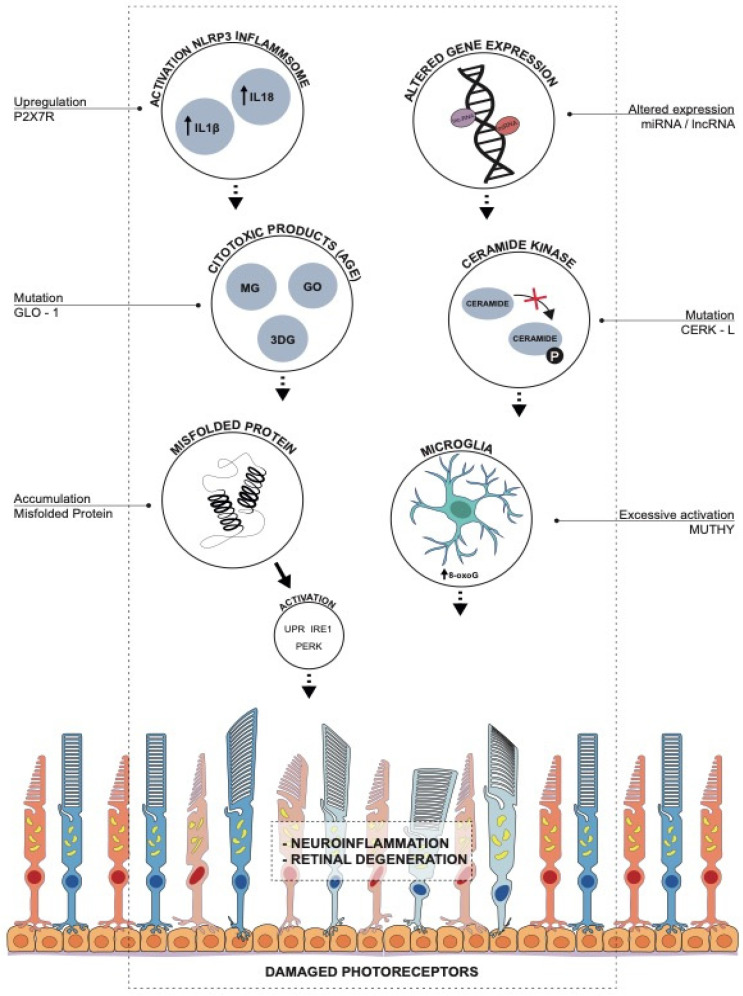
Main molecular mechanisms occurring during oxidative stress in RP.

The molecular mechanisms related to oxidative stress occurring in retinitis pigmentosa (Figure 1) play a central role in its pathogenesis and progression. The burden of the analysed pathways demonstrate that oxidative microglial activation may trigger the vicious cycle of non-resolved neuroinflammation and degeneration in RP, suggesting that the microglia may be a key target of oxidative stress in RP. Failure of the endogenous mechanisms to overcome oxidative stress leads to an accelerated progression of retinal neurodegeneration.

This analytic review aimed to highlight possible therapeutic targets inside the different pathogenetic mechanisms that induce the formation of ROS and potentially slow down the evolution of the disease over time.

## Figures and Tables

**Table 1:** Altered pathway involved in endogenous antioxidant defence and their effects on retinal tissue.

	Pathway Involved	Effects	Association with RP	References
*CHRONIC ACTIVATION OF PERK AND IRE1*	Misfolded proteins: UPR activation	Activation of pro-apoptotic programs Pro-inflammatory signalling Dysfunctional autophagy, free cytosolic Ca^2+^	Altered protein synthesis rate in the retina and retinal degeneration	[4,5]
*MUTYH MUTATION*	DNA repair	Formation of single-strand breaks (SSBs) of DNA Disturbed homeostasis and cell death Oxidative microglial activation	Retinal degeneration and neuroinflammation in RP	[16,18,20]
*CERKL MUTATION*	Oxidative stress protection	Activation of pro-apoptotic programs	Accelerated progression of retinal neurodegeneration.	[21,26,27]
*GLO1 MUTATION*	Detoxification of cytotoxic products of glycolysis	Inactivation of antioxidant enzymes (glutathione peroxidase and SOD enzymes)	Hyperinflammation and permanent tissue damage Vascular dysfunction Altered transduction pathways and genetic expression in EPR	[28,30,33,38]

**Table 2:** *GLO1*-related genes mutations: RPE dysfunction and photoreceptors damage.

Gene	Effects of Mutation	Consequences	References
*AUTS2*, *ANKH*	Alteration of actin filament structure and activity	RPE apoptosis	[41]
*SIK3*, *IPO3*, *MRPS33*	Dysregulation of energy metabolism and translation machinery	Cell death	[56,58,59]
*ARHGAP2*, *PTPN13*	Alteration of cell migration and proliferation Modification of cell polarity, cell adhesion, Golgi regulation Impairment of intracellular trafficking and glucose homeostasis	RPE apoptosis	[42,43,44,45]
*FMNL2*	Alteration of actin polymerization and organization of the cytoskeleton	Alteration of vesicular trafficking of RPE cells	[46,47]
*UBC*, *MYO18A*, *EPS15*, *ANKH*	Impairment of intracellular transport processes	Influence of vesicular trafficking of RPE cells (essential for POS renewal and visual cycle intermediate regeneration) AGE accumulation	[48,49,50,51]
*RFFL*, *FBXW2*, *CAND1*	ER stress Accumulation of misfolded proteins AGEs and ROS production	Cell death Alteration of cellular respiration process Impairment of glycolytic metabolism in RPE cells.	[52,53,54]
*SIK3*	Defect in energy metabolism Decrease of mitochondrial respiration Up-regulation of autophagy	Cellular antioxidant mechanisms impairment Alteration of cellular respiration process	[56,57]
*IPO7*	Ribosomal biogenesis stress Nucleolar morphology changes	p53-dependent growth arrest	[58]
*MRPS33*, *MORC4*, *MCPH1*, *NFIA*, *CTIF*, *LMBRD1*	Damage of mitochondrial protein synthesis Arrests in DNA damage repair Impairment of mitotic exit and cell differentiation Impairment of mRNA and protein quality control Alteration of transport and metabolism of cobalamin	RPE apoptosis and retinal degeneration Decreased distribution of cyanocobalamin (vitamin B12) from blood to retina	[59,62,63,64]

## Abbreviations

3-DG	3-DeoxyGlucosone
8-oxoG	8oxoGuanine
ACACA	Acetyl-CoA Carboxylase Alpha
AGEs	Advanced Glycation End products
AIPL1	Aryl Hydrocarbon Receptor Interacting Protein Like 1
AKT1	*AKT* Serine/Threonine Kinase 1
ALEs	Advanced lipoxidation End-products
ANKH	Progressive ankylosis protein homolog
ARF	Alternate Reading Frame
ARHGAP21	Rho GTPase Activating Protein 21
AUTS2	Cytoplasmatic activator of transcription and developmental regulator
BDNF-AS	Brain-derived neurotrophic factor Antisense
BER	Base Exicision Repair
C3	Complement Component 3
CAND1	Cullin-associated NEDD8-dissociated protein 1
CAP1	Cyclase Associated Actin Cytoskeleton Regulatory Protein 1
CERKL	Ceramide Kinase Like
CR3	Complement Receptor 3
CRD	Cone-Rode Dystrophy
CREB	cAMP response element-binding protein
CRNDE	Colorectal Neoplasia Differentially Expressed
CSF1R	Colony Stimulator Factor 1 Receptor (PLX5622)
CTIF	Cap Binding Complex Dependent Translation Initiation Factor
CYTOR	Cytoskeleton Regulator RNA
EPS15	Epidermal Growth Factor Receptor Pathway Substrate 15
FBXW2	F-box/WD repeat-containing protein 2
FMNL2	Formin Like Protein 2
FMR1	FMRP Translational Regulator 1
GAPDH	Glyceraldehyde 3-phosphate Dehydrogenase
GLO1	Glyoxalase 1
GO	Glyoxal
hMTH1	Human MutT Homologue
HSP	Heat Shock Protein
iBRB	Inner Blood-Retinal Barrier
IGF1	insulin-like growth factor-1
IL-18	*Interleukin-18*
IL-1β	*Interleukin-1β*
IPO3	Transportin-3
IPO7	Importin 7
IRE1	Inositol-Requiring Enzyme 1
KLHL7	Kelch Like Family Member 7
LC3-II	Lipidated form of LC3 (Microtubule-associated protein 1A/1B-light chain 3)
LMBRD1	Limb Development Membrane Protein Domain 1
lnc-RNA	long non-coding-RNA
MCPH	Microcephalin
MG	MethylGlyoxal
MIR31HG	MicroRNA 31 Host Gene
miRNA	microRNA
miRNome	Murine miRNA
MNX	Motor Neuron And Pancreas Homeobox 1
MNX-AS1	antisense transcript of MNX1
MORC 4	MORC family CW-type zinc finger protein 4
MRPS33	28S ribosomal protein S33
mTOR	Mammalian target of Rapamycin
MUTYH	mutY DNA glycosylase
MYO18A	Myosin XVIIIA
NFIA	Nuclear Factor I A
NLS	Nuclear Localization Signals
NPC	Nuclear Pore Complex
ONL	Outer Nuclear Layer
OS	Oxidative Stress
P2X7R	P2X 7 receptor
PARP	Poly(ADP-ribose) polymerase
PEDF	Pigment Epithelium-derived Factor
PERK	PKR-like Endoplasmic Reticulum Kinase
PI3K	Phosphoinositide 3 Kinase
PINK1	PTEN-induced kinase 1
POS	Photoreceptor Outer Segment
PPAR	peroxisome proliferator-activated receptor
PRC1	Polycomb Complex 1
PTEN	Phosphatase and tensin homolog
PTPN13	Protein Tyrosine Phosphatase Non-Receptor type 13
RAC1	Rac Family Small GTPase 1
RDH11	Retinol Dehydrogenase 11
RE	Rough Endoplasmic reticulum
RFFL	E3 ubiquitin-protein ligase rififylin
ROS	Reactive Oxygen Species
RPE	Retinal Pigment Epithelium
SIK3	SIK Family Kinase 3
SL	SphingoLipid
SOD	Superoxide Dismutase
SRGAP1	Slit-Robo Rho GTPase Activating Protein 1
SSB	Single Strand Breaks
TUG1	Taurine Up-Regulated 1
UBC	Ubiquitin C
UPR	Unfolded Protein Response
USH1G	Usher syndrome type-1G protein
VEGF	Vascular Endothelial Growth Factor
VIM	Vimentin
